# RNA networks of lysosomal-related biomarkers in Parkinson’s disease and their correlations with freezing of gait-associated genes

**DOI:** 10.3389/fgene.2026.1632163

**Published:** 2026-01-28

**Authors:** Zheng Qibin, Lin Lin, Chen Yibiao, Lin Peng, Wang Huiqing, Su Daoqing, Yu Lianghong

**Affiliations:** 1 Department of Neurosurgery, National Regional Medical Center, The First Affiliated Hospital, Binhai Campus of the First Affiliated Hospital, Fujian Medical University, Fuzhou, Fujian, China; 2 Department of Neurosurgery, Minnan Branch of The First Affiliated Hospital, Fujian Medical University, Quanzhou, Fujian, China; 3 Fujian Provincial Clinical Research Center for Neurological Diseases, The First Affiliated Hospital, Fujian Medical University, Fuzhou, China; 4 Department of Neurology, The Second Affiliated Hospital of Fujian Medical University, Quanzhou, China; 5 Department of Pain Management, First Affiliated Hospital, Fujian Medical University, Fuzhou, China; 6 Pain Research Institute, School of Basic Medical Sciences, Fujian Medical University, Fuzhou, China; 7 Neurosurgery Department of Shandong First Medical University Affiliated Central Hospital, Jinan, China; 8 Center for Movement Disorders and Neuropathic Pain, BCI Patient Room, Xuanwu Jinan Hospital, Jinan, China

**Keywords:** freezing of gait, immune infilitration, lysosome, miRNAs, molecular regulatory network, Parkinson’s disease

## Abstract

**Background:**

Parkinson’s disease (PD) is influenced by various factors, with lysosome function playing a critical role. However, the specific involvement of lysosome-related genes (LRGs) in PD remains unclear.

**Objective:**

This study aims to identify biomarkers specific to PD that exhibit robust disease prediction capabilities.

**Methods:**

Datasets for patients with PD, LRGs, and inflammation-related genes (IRGs) were retrieved from online databases. miRNAs and mRNAs within key modules were selected through Weighted Gene Co-expression Network Analysis (WGCNA), revealing strong associations with PD. A miRNA-mRNA network was constructed based on highly correlated PD-related LRGs (PD-LRGs) and miRNAs within these modules. Candidate genes were identified by intersecting target genes, differentially expressed genes (DEGs), PD-LRGs, and module-associated mRNAs. Machine learning and expression validation were employed to confirm these biomarkers. A nomogram was established, and its diagnostic performance was evaluated using a confusion matrix. Drug predictions were conducted based on these biomarkers. Spearman’s correlation analyses were performed to assess the relationship between IRGs, freezing of gait (FOG)-related genes, and biomarkers. Molecular regulatory networks were constructed using datasets and online resources. Finally, clinical samples were collected for quantitative PCR (qPCR) validation of biomarker expression.

**Results:**

Key modules related to PD were identified, comprising 190 miRNAs and 7,633 mRNAs. A miRNA-mRNA network was constructed based on 55 PD-LRGs and 181 miRNAs, resulting in the identification of 26 candidate genes strongly linked to lysosomal function. *FGD4* and *MAN2B1* were selected as biomarkers, and a gene expression-based risk prediction table was created. These biomarkers were significantly correlated with IRGs and several FOG-related genes. Gene localization analysis revealed that *FGD4* and LRRK2, both critical to the FOG pathway, are located on chromosome 12. Drug prediction revealed that Tetrachlorodibenzodioxin and bisphenol A target both *FGD4* and *MAN2B1*. qPCR analysis confirmed that *FGD4* and *MAN2B1* expression levels were significantly higher in patients with PD compared to healthy controls (*p* < 0.05).

**Conclusion:**

*FGD4* and *MAN2B1* act as lysosomal biomarkers associated with PD and exhibit strong correlations with genes involved in PD-related freezing of gait. This study offers novel insights into PD diagnosis.

## 1 Introduction

Parkinson’s disease (PD), the second most prevalent neurodegenerative disorder after Alzheimer’s disease (AD), affects approximately 7 million individuals worldwide (Chi et al., 2019). It is characterized by the progressive degeneration of dopaminergic (DA) neurons in the substantia nigra, leading to clinical manifestations such as bradykinesia, myotonia, resting tremor, and postural instability (Samii et al., 2004; Schneider et al., 2017). The exact etiology of PD remains unclear, likely resulting from a complex interplay of genetic and environmental factors (Zhang et al., 2021). Due to its intricate pathogenesis, treatment primarily focuses on symptomatic management, such as dopamine replacement therapy (Antony et al., 2013). However, disabling axial symptoms, particularly postural instability and freezing of gait (FOG), often remain resistant to both medication and surgical interventions (Boonstra et al., 2008; Nieuwboer and Giladi, 2013). Recently, molecular biomarkers have emerged as promising tools for PD diagnosis (Chen-Plotkin et al., 2018). Consequently, there is a pressing need to explore novel methods for early detection and more precise treatments, especially for patients with refractory FOG.

Lysosomes, traditionally known as organelles responsible for cellular digestion, degradation, and recycling of metabolic waste, have recently been shown to play critical roles in cellular metabolism, proliferation, differentiation, apoptosis, immunity, nutrient sensing, protein regulation, and metabolic signaling (Trivedi et al., 2020). The involvement of lysosomal function in PD has been confirmed through both functional and genetic studies (Senkevich and Gan-Or, 2020). PD is characterized by the degeneration of DA neurons and the accumulation of Lewy bodies, primarily composed of misfolded and aggregated α-synuclein proteins (Fares et al., 2021). Lysosomes are the primary site for the degradation of aggregated α-synuclein (Lee et al., 2004; Vogiatzi et al., 2008; Mak et al., 2010). Mutations in lysosomal genes contribute to elevated levels of α-synuclein or its increased tendency to aggregate, thereby enhancing the genetic risk of PD (Ibáñez et al., 2004; Pihlstrøm et al., 2018). Cathepsin B (catB), a proteolytic enzyme of the cysteine cathepsin family with both endopeptidase and exopeptidase activities, is typically localized in the lysosomal cavity (Stoka et al., 2016). Knockdown of the PD risk gene TMEM175 disrupts lysosomal pH and impairs catB activity (Jinn et al., 2017; Hu et al., 2022). Additionally, mutations in *LRRK2*, a major cause of familial PD, have been shown to inhibit catB expression or activity within lysosomes (Henry et al., 2015; Yadavalli and Ferguson, 2023). Lysosomal dysfunction caused by mutations in these genes can exacerbate the accumulation of α-synuclein in the brain, potentially accelerating the onset of PD (Blumenreich et al., 2020). Although several studies have linked PD onset and progression to lysosome-related genes (LRGs), the precise genetic mechanisms remain poorly understood. Investigating LRGs holds promise for identifying PD biomarkers, aiding in the development of preventive strategies, early diagnosis, and more effective management, while also enhancing the understanding of underlying mechanisms and reducing risks.

In recent years, high-throughput technologies have made significant advances in PD biomarker research, yet critical limitations remain. Current studies primarily follow three directions: First, numerous blood or cerebrospinal fluid transcriptomic studies have screened candidate gene profiles through differential expression analysis. However, over 70% of identified markers lack independent validation (Chen-Plotkin et al., 2018), limiting their clinical translational value. Second, explorations of biomarkers related to specific pathways such as oxidative stress (Zhu et al., 2025) and copper metabolism (Lin et al., 2024) have revealed some pathological mechanisms but have failed to systematically integrate interactions between multiple pathways. Third, while immune-related gene signatures can distinguish PD from healthy controls, they often lack specificity for early diagnosis and disease subtypes (Baird et al., 2019). More notably, although genome-wide association studies have consistently confirmed significant associations between lysosome-related genes (LRGs, such as *TMEM175*, *LRRK2*, and *GBA*) and PD risk (Nalls et al., 2019), functional studies targeting the complete LRG set remain scarce, and their predictive value for PD diagnosis and their links to core motor symptoms have yet to be clarified. Addressing this critical gap, this study, for the first time, systematically identifies lysosome-related biomarkers highly associated with PD phenotypes by integrating weighted gene co-expression network analysis (WGCNA), machine learning algorithms, dual-cohort cross-validation, and qPCR confirmation. It further constructs miRNA-mRNA regulatory networks and performs multidimensional correlation analyses linking the identified biomarkers with FOG-related genes and immune cell infiltration. This work establishes an integrated analytical framework of “lysosomal dysfunction–immune dysregulation–motor symptoms,” offering a novel strategy for early PD diagnosis and precision treatment that combines mechanistic depth with clinical applicability.

## 2 Materials and methods

### 2.1 Data selection and preprocessing

In this study, three transcriptomic datasets were collected from the Gene Expression Omnibus (GEO) database (https://www.ncbi.nlm.nih.gov/geo/), including GSE100054, GSE99039, and GSE16658. All disease samples were diagnosed as PD. The lncRNA, miRNA, and mRNA profiles of GSE100054 (GPL23126) included peripheral blood mononuclear cells (PBMCs) from 10 patients with PD and 9 normal controls (Miki et al., 2018). The mRNA profile of GSE99039 (GPL570) contained whole blood samples from 205 patients with idiopathic PD and 233 normal controls (Shamir et al., 2017). Additionally, the miRNA profile of GSE16658 (GPL7722) included PBMCs from 19 patients with PD and 13 normal controls (Martins et al., 2011). A total of 144 LRGs and 200 inflammation-related genes (IRGs) were collected from the published literature and the Molecular Signatures Database (MSigDB, https://www.gsea-msigdb.org/gsea/msigdb, HALLMARK_INFLAMMATORY_R ESPONSE.v2022.1), respectively (Supplementary Table S1; Supplementary Table S2) (Vairo et al., 2017).

All datasets were independent GEO datasets, and the following preprocessing steps were performed before analysis: (1) Six quantiles (0%, 25%, 50%, 75%, 99%, 100%) were calculated for the expression data; (2) If the 99th percentile exceeded 100, indicating the presence of large values, a log_2_ transformation was performed; (3) If the difference between the maximum and minimum values was greater than 50 and the lower quartile (25%) was greater than 0, indicating a large data range with no negative values, a log_2_ transformation was applied; (4) If the lower quartile (25%) was between 0 and 1, and the upper quartile (75%) was between 1 and 2, indicating that the data were concentrated in a low range, a log_2_ transformation was carried out to enhance the differences. These preprocessing steps ensured the data followed a normal distribution.

### 2.2 Weighted gene Co-expression network analysis (WGCNA)

To identify miRNAs and mRNAs related to PD, WGCNA was conducted using the WGCNA (v. 1.71) package (Langfelder and Horvath, 2008). Initially, hierarchical clustering analysis was performed on all samples from the GSE100054 dataset based on Euclidean distance using the complete linkage method, and abnormal samples were removed based on clustering results. Next, the soft threshold (β) was determined when the goodness of fit *R* ^2^ reached 0.85, at which point the network approximated a scale-free distribution. All miRNAs and mRNAs were then clustered into several modules (minModuleSize = 100, MEDissThres = 0.3). Modules associated with PD were selected as critical modules (|correlation (cor)| > 0.30, *p* < 0.05), and the miRNAs and mRNAs in these critical modules were used for further analysis.

### 2.3 Acquisition of LRGs in PD (PD-LRGs) and construction of miRNA-mRNA network

The intersection of mRNAs in critical modules and LRGs was considered as PD-LRGs. Subsequently, Spearman’s correlation analysis between PD-LRGs and miRNAs in the critical modules was performed (cor < −0.30, *p* < 0.05), and based on these results, a miRNA-mRNA network was constructed.

### 2.4 Identification of candidate genes

Differentially expressed genes (DEGs) between patients with PD and normal controls were identified in GSE100054 using the limma (v. 3.52.4) package (|log_2_Fold Change (FG)| ≥ 0.5, *p* < 0.05) (Ritchie et al., 2015). A volcano plot of the DEGs was generated using the ggplot2 (v. 3.3.6) package (Maag, 2018). The target genes of miRNAs in critical modules were retrieved from the miRNet (https://www.mirnet.ca/) and miRWalk (http://129.206.7.150/) databases. The candidate genes were identified by intersecting DEGs, target genes from miRNet, target genes from miRWalk, PD-LRGs, and mRNAs in critical modules. A heatmap of the candidate genes was generated using the pheatmap (v. 1.0.12) package (Gu et al., 2016).

### 2.5 Function enrichment analysis of candidate genes

To explore the potential functions and pathways of the candidate genes, Gene Ontology (GO) and Kyoto Encyclopedia of Genes and Genomes (KEGG) enrichment analyses were conducted using the clusterProfiler (v. 4.6.0) package (adj.*p* < 0.05) (Yu et al., 2012). The GO analysis categorized genes into three distinct categories: cellular components (CC), molecular functions (MF), and biological processes (BP). The top 5 GO terms with the highest significance in each category and the top 5 KEGG pathways with the highest significance were selected for presentation.

### 2.6 Identification of biomarkers in patients with PD

At the protein level, a protein-protein interaction (PPI) network of the candidate genes was constructed based on the STRING database (https://cn.string-db.org/) (medium confidence ≥0.4). Isolated nodes (degree = 0) were filtered out to refine the network. Subsequently, Least Absolute Shrinkage and Selection Operator (LASSO) regression analysis was performed to identify hub genes in GSE100054 using the glmnet (v 4.1-6) package (α = 1, nfolds = 3, family = “binomial”) (Engebretsen and Bohlin, 2019). The optimal λ values (lambda.min and lambda.1se) were selected through cross-validation to balance the model complexity and prediction performance. Significant differences in expression levels between PD and control samples were observed in GSE100054 and GSE99039, and genes with consistent expression trends across these datasets were selected as biomarkers (*p* < 0.05). A logistic regression model was then constructed based on the biomarkers, and a nomogram was generated for visualization using the rms (v. 6.5-0) package (https://CRAN.R-project.org/package=rms). Specifically, the lrm function of the rms package was used for model fitting, with x = TRUE and y = TRUE specified to store the design matrix and response variable, and maxit = 1000 set to ensure convergence. A nomogram was plotted via the nomogram function, where the risk probability transformation function was defined as fun = 1/(1+exp (-x)) and probability scales ranging from 0.01 to 0.99 were displayed. Finally, a standard logistic regression model was constructed using the glm function with family = “binomial”. A confusion matrix was created to evaluate the prediction accuracy of the model using the caret (v. 6.0–36) package (https://doi.org/10.18637/jss.v028.i05).

### 2.7 Evaluation of infiltrating immune cells

To assess immune cell infiltration in GSE100054, the estimated proportions of 28 immune cell types were calculated using the single-sample gene set enrichment analysis (ssGSEA) algorithm from the GSVA (v. 1.44.5) package (Hänzelmann et al., 2013). The differences in immune cell content between PD and normal controls were compared using the Wilcoxon test (*p* < 0.05). Spearman’s correlation analysis was performed between biomarkers and immune cells (|cor| > 0.30, *p* < 0.05).

### 2.8 Spearman’s correlation of DE-IRGs and FOG with biomarkers, respectively

Differentially expressed IRGs (DE-IRGs) were identified by overlapping the 200 IRGs and DEGs in GSE100054, and the expression levels of 36 DE-IRGs were analyzed. Chromosome mapping of the FOG genes and biomarkers was visualized using the “RCircos (v. 1.2.2)” R package (Zhang et al., 2013). To further explore the relationship between DE-IRGs and the 9 FOG-related genes (*LRRK2*, *NEFL*, *GFAP*, *DRD2*, *ANKK1*, *COMT*, *DHCR7*, *NADSYN1*, and *CYP2R1*) with biomarkers in PD, Spearman’s correlation analysis was conducted (|cor| > 0.30, *p* < 0.05).

### 2.9 Prediction of transcription factors (TFs) and construction of lncRNA-miRNA-mRNA network

To explore the molecular regulatory relationships of the biomarkers, TFs were predicted using the PASTAA database (http://trap.molgen.mpg.de/PASTAA.htm) based on the biomarkers. The TF with the highest association score (*p* < 0.05) was selected, and its binding sites were visualized using the JASPAR database (https://jaspar.genereg.net/). Hub miRNAs were selected based on the following criteria: (1) miRNAs associated with biomarkers in GSE100054 and GSE16658, (2) consistent expression trends between GSE100054 and GSE16658, and (3) opposite expression trends compared to biomarkers. The lncRNAs were then predicted based on the hub miRNAs using the Starbase database (http://starbase.sysu.edu.cn/) (clipExpNum ≥5). Finally, an lncRNA-miRNA-mRNA network was constructed based on the selected lncRNAs, hub miRNAs, and biomarkers.

### 2.10 Potential drug prediction

To identify potential drugs targeting the biomarkers for patients with PD, the Drug-Gene Interaction Database (DGIdb, https://ctdbase.org/) and the CTD database were utilized. Drugs with an interaction score ≥4 were considered key drugs, and a drug-gene network was constructed using Cytoscape (v. 3.7.2) software (Shannon et al., 2003).

### 2.11 Clinic specimens, RNA extraction, and quantitative PCR (qPCR)

For experimental validation, blood samples from 5 patients with PD and 5 healthy controls were collected at the First Affiliated Hospital of Fujian Medical University. The study was approved by the Ethics Committee of the First Affiliated Hospital of Fujian Medical University. Total RNA was extracted from the specimens using TRIzol Reagent (Ambion, Shanghai, China). Subsequently, 0.1 ng to 5 µg of RNA was used to synthesize complementary DNA (cDNA) with the SweScript First Strand cDNA Synthesis Kit (Servicebio, Wuhan, China). Primer sequences for the biomarkers were synthesized by Beijing Tsingke Biotech Co., Ltd. (Beijing, China) (Supplementary Table S3). Quantitative PCR (qPCR) was conducted with the CFX96™ Real-Time PCR Detection System (BIO-RAD, U.S.A.) in 40 cycles. The relative expression levels of biomarkers were calculated using the 2^−ΔΔCT^ method (Rao et al., 2013), with all samples run in triplicate.

### 2.12 Statistical analysis

All statistical analyses were performed using R (v. 4.2.3) software. Spearman’s correlation was used to analyze associations, and a *p*-value <0.05 was considered statistically significant (two-tailed).

## 3 Results

### 3.1 A total of 7,823 miRNAs and mRNAs in critical modules related to PD were determined

**FIGURE 1:**
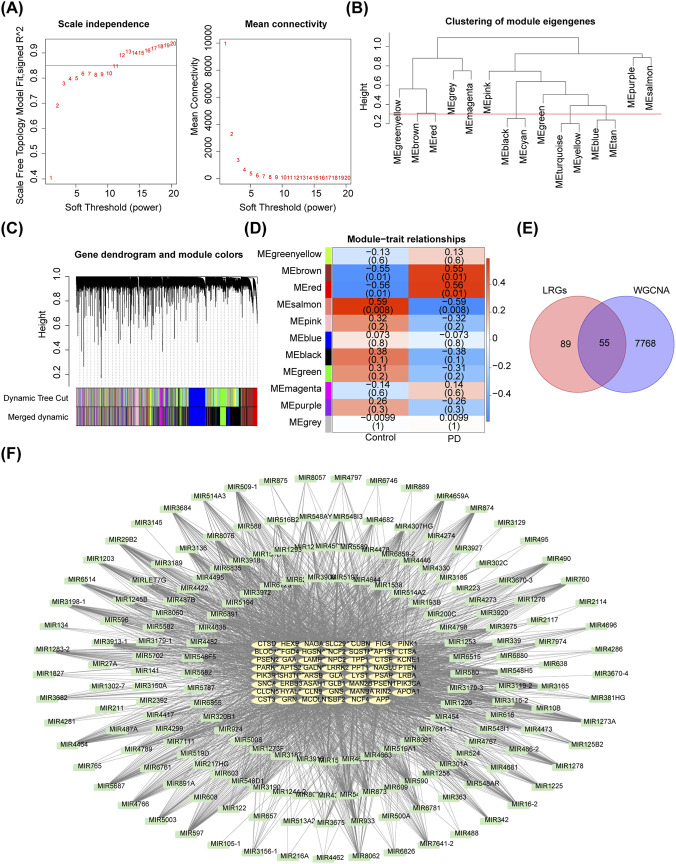
Constructive process of PD-LRGs-miRNA network. **(A)** Power law distribution and stable trend of average connection of the data for analysis. **(B)** Clustering of module eigengenes. **(C)** Gene dendrogram of the 13 modules after merging similar modules. **(D)** MEsalmon, MEred, and MEbrown screened as critical modules from Module-trait relationship analyze. **(E)** 55 intersecting mRNAs were obtained as PD-LRGs. **(F)** miRNA-mRNA network of PD-LRGs,including 55 PD-LRGs and 181 miRNAs.

WGCNA was conducted to identify critical modules associated with PD. As shown in Supplementary Figure S1, the branch heights of all samples in the GSE100054 dataset were concentrated within the range of 80–180. No sample formed an independent branch, and the connection heights between samples were consistent with the overall distribution, suggesting that no samples needed to be excluded. Subsequently, 15 modules were identified based on a β value of 12 (Figures 1A,B). After merging similar modules, 13 modules remained for further analysis (Figure 1C). Three critical modules highly correlated with PD—MEsalmon, MEred, and MEbrown—were identified, comprising a total of 7,823 miRNAs and mRNAs (190 miRNAs and 7,633 mRNAs) with a correlation |cor| > 0.30 and *p* < 0.05 (Figure 1D; Supplementary Tables S4, S5). From these, 55 PD-related LRGs (PD-LRGs) were obtained (Figure 1E). The PD-LRGs-miRNA pairs were constructed, including 55 PD-LRGs and 181 miRNAs, with a correlation of cor < −0.30 and *p* < 0.05 (Figure 1F).

### 3.2 There were 26 candidate genes in GSE100054

**FIGURE 2:**
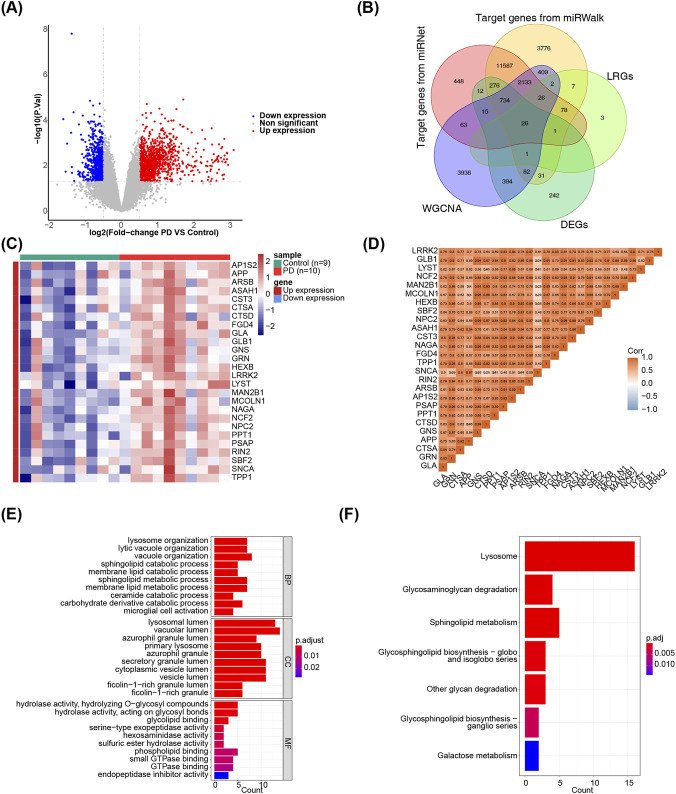
Identification of candidate genes. **(A)** DEGs between PD and normal controls **(B)** 26 candidate genes idenficated in training set **(C)** Expression levels of the candidate genes were all upregulated **(D)** Positive correlation of the candidate genes. Enrichment analyses of candidate genes by GO **(E)** and KEGG **(F)**.

A total of 1,814 DEGs between patients with PD and normal controls were identified in GSE100054, including 1,073 upregulated DEGs and 741 downregulated DEGs (Figure 2A; Supplementary Table S6). Based on the 190 miRNAs in the critical modules, 15,399 target genes were predicted using the miRNet database, and 19,169 target genes were identified in the miRWalk database (Supplementary Tables S7, S8). Subsequently, 26 candidate genes were selected for further analysis (Figure 2B). Notably, the expression levels of these 26 candidate genes were upregulated in PD samples (|log_2_FC| ≥ 0.5, *p* < 0.05) (Figure 2C), with most showing a significantly positive correlation (Figure 2D). The GO analysis identified 252 enriched GO terms, including lysosome organization, lytic vacuole organization, and vacuole organization. Seven KEGG pathways, such as lysosome, glycosaminoglycan degradation, and sphingolipid metabolism, were also significantly enriched (adj. *p* < 0.05) (Figures 2E,F; Supplementary Tables S9, S10).

### 3.3 FGD4 and MAN2B1 were biomarkers for the diagnosis of patients with PD

**FIGURE 3:**
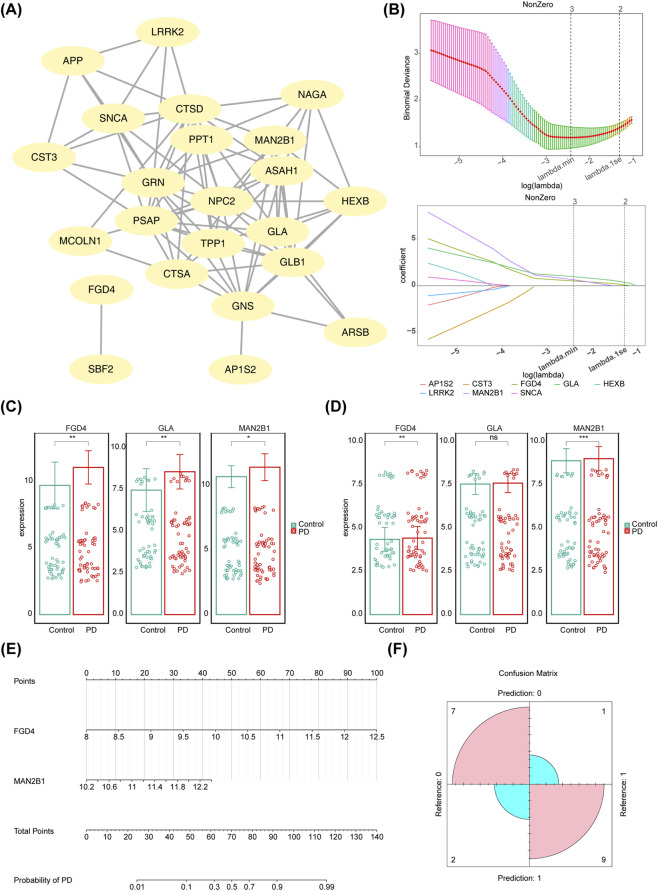
Identification of biomarkers for PD diagnosis. **(A)** Interaction of the 26 candidate genes. **(B)** 3 hub genes selected by Lasso analysis of candidate genes as biomarkers. Expression level of the biomarkers in training set **(C)** and Validation set **(D)**. **(E)** Nomogram model of the biomarkers for PD prediction. **(F)** Confusion matrix demonstrated outstanding predictive ability of the model.

A PPI network was constructed based on the 26 candidate genes, revealing three isolated candidate genes that had no interaction with other proteins (Figure 3A). The remaining 23 candidate genes underwent Lasso analysis, leading to the selection of three hub genes—*FGD4*, *GLA*, and *MAN2B1*—based on the optimal lambda value of 0.0875 (Figure 3B). *FGD4* and *MAN2B1* were further validated as biomarkers due to their significant expression levels (Figures 3C,D). Specifically, the expression of *FGD4* was significantly higher in the PD group compared to the control group in both GSE100054 (*p* = 0.0044) and GSE99039 (*p* = 0.0089). Likewise, *MAN2B1* expression was significantly higher in the PD group compared to controls in GSE100054 (*p* = 0.0133) and GSE99039 (*p* = 0.0014). Although *GLA* was upregulated in the PD group of GSE100054 (*p* = 0.0045), no significant difference was observed between the PD and control groups in GSE99039 (*p* = 0.0894). *FGD4* and *MAN2B1*, selected as biomarkers, were both highly expressed in the PD group. Finally, a nomogram model was constructed based on these biomarkers (*FGD4* and *MAN2B1*), and the confusion matrix demonstrated the model’s outstanding predictive performance (Figures 3E,F).

### 3.4 Spearman’s correlation analyses were performed between biomarkers and immune cells, IRGs, and FOG-related genes, respectively

**FIGURE 4:**
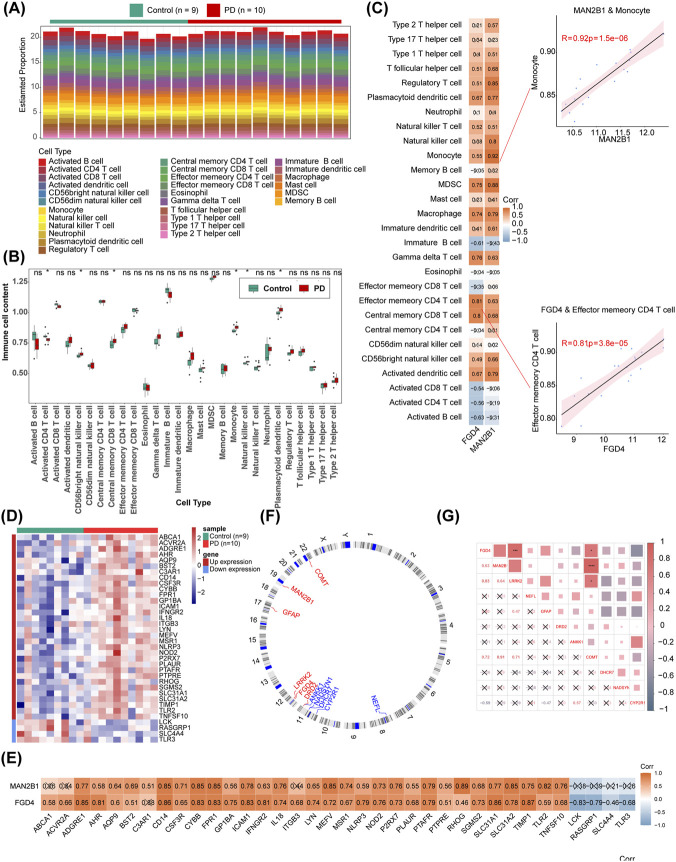
Correlation between biomarkers and immune cells, IRGs, and LRRK2. **(A)** Estiamted proportion of 28 immune cells by ss GSEA. **(B)** Content differences of infiltrating immune cells. **(C)** Correlation between biomarkers and immune cells. **(D)** Expression levels of IRGs, that 32 were upregulated and 4 downregulated. **(E)** Correlation between the 3 PD biomarkers and IRGs. **(F)** Correlations between LRRK2 and biomarkers. **(G)** Chromosome mapping of biomarkers and LRRK2.

The estimated proportions of 28 immune cell types are shown in Figure 4A. In the comparison between PD and normal controls in GSE100054, significant differences in immune cell infiltration were observed for 6 immune cell types: activated CD4 T cells, CD56 bright natural killer cells, central memory CD8 T cells, monocytes, natural killer cells, and plasmacytoid dendritic cells (Figure 4B). Spearman’s correlation analysis revealed that *MAN2B1* had the strongest correlation with monocytes (cor = 0.92, *p* < 0.001), while *FGD4* showed the strongest correlation with memory effector CD4 T cells (cor = 0.81, *p* < 0.001) (Figure 4C). In patients with PD from GSE100054, 36 DE-IRGs were identified, of which 32 were upregulated and 4 were downregulated (Figure 4D). Significant positive correlations were found between 29 DE-IRGs and *MAN2B1* (|cor| > 0.30, *p* < 0.05), with *MAN2B1* exhibiting the strongest correlation with *RHOG* (cor = 0.87, *p* < 0.05). All DE-IRGs, except *C3AR1*, were significantly correlated with *FGD4* (|cor| > 0.30, *p* < 0.05), with *FGD4* showing the strongest correlation with TIMP1 (cor = 0.87, *p* < 0.05) (Figure 4E). Additionally, among the DE-IRGs, LCK exhibited the strongest negative correlation with *FGD4* (cor = −0.83, *p* < 0.05), whereas no negative correlation was observed between MAN2B1 and any DE-IRGs. Furthermore, the chromosome distribution of biomarkers and the 9 FOG genes is illustrated in Figure 4F. Chromosome localization analysis revealed that *MAN2B1* is located on chromosome 19, while both *FGD4* and *LRRK2* are located on chromosome 12. Significant correlations were found between *FGD4* and LRRK2 (cor = 0.83, *p* < 0.05), as well as between *FGD4* and *COMT* (cor = 0.72, *p* < 0.05). *MAN2B1* was also significantly correlated with *COMT* (cor = 0.91, *p* < 0.05), though no significant correlation was observed between *FGD4* and *MAN2B1* (cor = 0.63, *p* > 0.05) (Figure 4G).

### 3.5 Molecular regulatory and drug-gene networks were built

**FIGURE 5:**
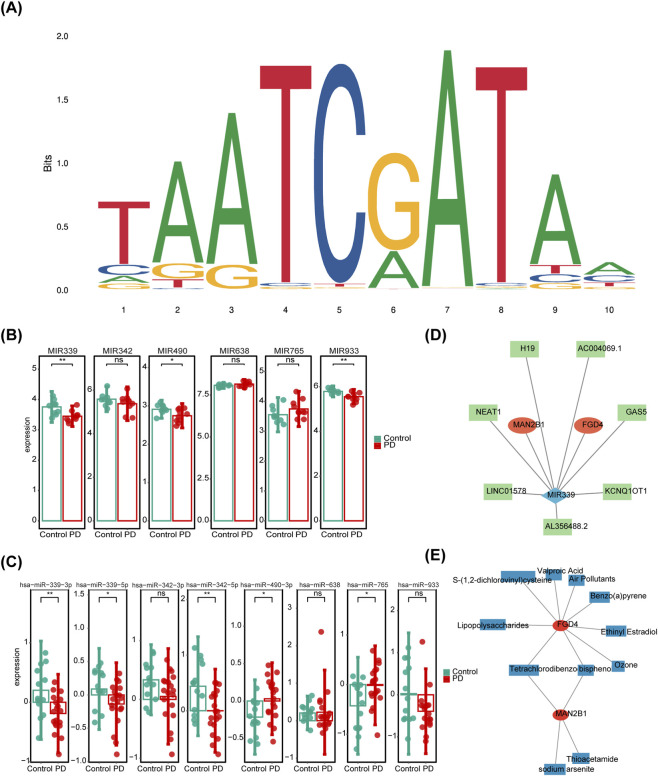
**(A)** Binding site of Cutl1, TF with the highest association score. MIR339, MIR342, and MIR933 selected as Hub miRNAs in GSE100054 **(B)** and GSE16658 **(C)**. **(D)** lncRNA-miRNA-mRNA network. **(E)** Visualization of drug prediction results.

In the PASTAA database, 32 TFs related to the biomarkers were predicted (Supplementary Table S11). The TF with the highest association score was Cutl1, and its binding site is shown in Figure 5A. Additionally, three hub miRNAs—MIR339, MIR342, and MIR933—were selected (Figures 5B,C). Hub miRNAs were selected based on their consistent dysregulation patterns across both the discovery cohort (GSE100054) and an independent miRNA cohort (GSE16658). Based on these hub miRNAs, 19 lncRNAs were predicted using the Starbase database, and a lncRNA-miRNA-mRNA network was constructed, including GAS5-MIR339-*FGD4*, XIST-MIR342-*FGD4*, and KCNQ1OT1-MIR339-*MAN2B1* (Figure 5D). Moreover, 11 drugs related to the biomarkers were predicted, with Tetrachlorodibenzodioxin and bisphenol A identified as common drugs targeting both *FGD4* and *MAN2B1* (Figure 5E).

### 3.6 FGD4 and MAN2B1 were highly expressed in patients with PD

**FIGURE 6:**
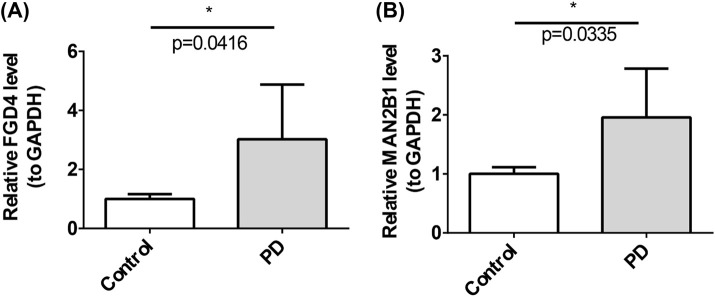
Expression level of PD biomarks in Clinic specimens by qPCR.

In patients with PD, the expression levels of *FGD4* (*p* = 0.0416) and *MAN2B1* (*p* = 0.0335) were significantly higher than those in healthy controls (Figures 6A,B).

## 4 Discussion

PD is a complex neurodegenerative disorder in which the pathogenesis involves an interplay between genetic and environmental factors, leading to dysregulation of essential biological processes such as lysosomal dysfunction. This dysfunction impairs α-synuclein degradation and accelerates DA neuron death (Kalia and Lang, 2015; Van Veen et al., 2020). Research has revealed significant overlap between LRGs and PD pathogenic genes, highlighting the urgent need for population-based LRG screening and novel PD biomarkers (Robak et al., 2017). This study integrates bioinformatics and experimental validation to identify two lysosomal function-related biomarkers in PD and elucidates their clinical significance and regulatory networks. Specifically, *FGD4* and *MAN2B1* consistently demonstrated robust diagnostic performance across dual cohorts (AUC >0.8), with their elevated expression in peripheral blood further confirmed by qPCR. More importantly, these two biomarkers are associated with FOG, a core motor symptom of PD. *FGD4* is co-localized with *LRRK2* on chromosome 12 and shows a significant positive correlation, while *MAN2B1* cooperatively regulates the dopamine metabolism pathway with *COMT*. Concurrently, immune infiltration analysis revealed their differential regulatory roles in the immune microenvironment: *MAN2B1* is strongly associated with monocytes, whereas *FGD4* shows a strong correlation with activated memory effector CD4^+^ T cells, thereby establishing a “lysosome-immune” interaction axis. Furthermore, this study predicted compounds such as Tetrachlorodibenzodioxin that can target these two biomarkers. Collectively, these multidimensional findings support lysosomal dysfunction as a novel target for early diagnosis and progression prediction in PD.

Focusing on the close relationship between lysosomal function and PD, this study highlights two critical biomarkers: *FGD4* and *MAN2B1*. *FGD4* (FYVE, RhoGEF, and PH domain-containing 4) is a protein that regulates the cytoskeleton and cell shape (Obaishi et al., 1998; Delague et al., 2007). Aberrant expression of *FGD4* disrupts the polarity, proliferation, and morphology of myelin sheaths, impairing nerve conduction (Obaishi et al., 1998; Ono et al., 2000). Moreover, *FGD4* is implicated in lysosomal encapsulation, endocytosis, and degradation processes (Delague et al., 2007). *MAN2B1*, another key biomarker, is closely linked to lysosomal accumulation responses (Wood et al., 2013), with proteomic analysis of cerebrospinal fluid identifying it as a potential PD biomarker (Karayel et al., 2022). Both *FGD4* and *MAN2B1* contribute to lysosomal membrane stability, endocytosis, and degradation. Overexpression of these genes may result in excessive protease release, autophagy system instability, or inflammatory response activation (Taylor et al., 2018). Further drug prediction analysis in this study indicates that Tetrachlorodibenzodioxin and Bisphenol A could intervene with *FGD4* and *MAN2B1*, providing novel therapeutic references for PD treatment. Bisphenol A has been shown to regulate autophagy through pathways such as AKT-mTOR (Zhang et al., 2023), while Tetrachlorodibenzodioxin, an immunosuppressive compound (Dooley and Holsapple, 1988), can reduce inflammatory cytokines like IFN-γ (Ciftci and Ozdemir, 2011). Tetrachlorodibenzodioxin may also inhibit the autophagy system’s phagocytosis-degradation function (Podechard et al., 2009). Therefore, these two predicted drugs are essential for targeting the immune-autophagy pathways associated with *FGD4* and *MAN2B1* in patients with PD. In conclusion, this study identified two LRGs with differential expression in patients with PD, predicting alterations in various lysosomal autophagy functions. These findings provide new insights into the pathogenic mechanisms related to lysosomal damage in PD, opening avenues for further exploration.

miRNAs have become a central focus in biological research, with several miRNAs linked to DEGs in various diseases. This study identified MIR342, MIR339, and MIR933 for the first time as miRNAs significantly associated with key biomarkers of PD-LRGs. MIR342 is known to be involved in telomerase activity and has a strong association with neurodegenerative diseases (Likonen et al., 2022). MIR339 has been recognized as a biomarker in atypical PD syndrome (Bougea, 2022), while MIR933 has been implicated in AD, where it interferes with nerve growth factor translation, leading to neuroinflammation (Dias et al., 2018). Notably, MIR342-3p promotes autophagy by inhibiting MAP1LC3B (Zhang et al., 2020), MIR339-5p significantly affects the phagocytic and degradative functions of immune cells (Hakimzadeh et al., 2017), and the upregulation of MIR933 has been shown to induce autophagy dysregulation (Mohammadi et al., 2018). In summary, this is the first study to identify the critical roles of multiple miRNAs in PD, providing new avenues for exploring the genetic pathogenic factors of the disease.

Immune cell infiltration analysis, combined with the overlay analysis of DE-IRGs for each biomarker, revealed that MAN2B1 was most strongly associated with monocyte infiltration. A strong positive correlation was found between MAN2B1 and RHOG. Upregulation of *MAN2B1* causes functional divergence in lysosomal α-mannosidase, affecting glycoconjugate modifications, which may facilitate monocyte infiltration by binding to the cell membrane (Urbanelli et al., 2011). RHOG, in turn, integrates multiple receptor signals during the phagocytic process of monocytes/macrophages (Tzircotis et al., 2011). Another analysis revealed that memory effector CD4 T cell infiltration was most strongly associated with *FGD4*, showing a strong positive correlation between *FGD4* and TIMP1. It is well established that α-synuclein accumulation in patients with PD acts as a primary antigen for memory effector CD4 T cells (Christiansen et al., 2016), which are linked to TIMP1 expression (Ding et al., 2022). The dopamine receptors on these cells are closely associated with FOG (Kustrimovic et al., 2016; Diener et al., 2023). Based on these findings, the activation of memory effector CD4 T cells in PD may lead to the upregulation of *FGD4*, thereby enhancing macropinocytosis in lysosomal encapsulation, endocytosis, and degradation (Charpentier et al., 2020). This hypothesis establishes a link between the adaptive immune response and the regulation of lysosomal function in PD pathogenesis. However, the causal relationships and underlying molecular mechanisms need further validation through functional experiments.

This study found that *FGD4* expression is significantly positively correlated with *LRRK2. LRRK2* is a key gene regulating lysosomal phagocytosis and degradation, and its mutations can inhibit cathepsin activity (Yadavalli and Ferguson, 2023). We speculate that *FGD4*, as a cytoskeletal regulator, may functionally synergize with LRRK2 in the lysosomal protein clearance pathway or be co-transcriptionally regulated by influencing lysosomal membrane stability or endocytic efficiency (Abe et al., 2024; Hughes et al., 2025). Additionally, both *FGD4* and *MAN2B1* in this study showed positive correlations with *COMT*, which is involved in dopamine metabolism and whose genotype is associated with the severity of motor symptoms in PD (Williams-Gray et al., 2009). This correlation suggests that lysosomal dysfunction may exacerbate metabolic stress within dopaminergic neurons. We hypothesize that abnormal release of lysosomal proteases (such as cathepsin B) may affect the activity of dopamine-metabolizing enzymes, thereby synergizing with *COMT* to aggravate motor symptoms (Williams-Gray et al., 2009). Interestingly, *FGD4* was negatively correlated with *CYP2R1*, a gene involved in vitamin D metabolism, providing a potential molecular link to the clinical observation of vitamin D deficiency and increased fall risk in PD patients—namely, that lysosomal stress may interfere with the expression of genes related to vitamin D metabolism (Wang et al., 2010; Ding et al., 2013; Mahanty et al., 2019). In summary, this study reveals associations between *FGD4/MAN2B1* and FOG-related genes, suggesting that lysosomal dysfunction may engage in molecular crosstalk with PD motor symptoms, particularly FOG. These associations offer new clues for understanding the lysosome-neuroinflammation-motor regulation network in PD. It is important to note that these findings are derived from bioinformatics correlation analyses and have not yet been functionally validated for causality. Therefore, future studies should employ functional experiments—such as knocking down or overexpressing *FGD4/MAN2B1* in cellular or animal models and assessing their effects on the expression of *LRRK2* and *COMT*, as well as on lysosomal function and motor behavior—to verify whether these correlations possess a causal and mechanistic basis. In summary, this study identified *FGD4* and *MAN2B1* as biomarkers related to lysosomal dysfunction in PD, revealing their roles in disrupting transcriptional networks and contributing to the progression of FOG through neuroinflammatory cascades. These findings offer novel insights into the lysosomal-autophagy-immune axis in PD. However, several limitations must be acknowledged: First, due to sample size constraints, the gene modules initially identified through WGCNA may lack stability. Additionally, conducting WGCNA, differential expression analysis, and subsequent internal validation within the same discovery cohort raises the risk of circular analysis. Similarly, the relatively small sample size used for qPCR validation may limit the statistical validity and generalizability of our findings. So the results should be interpreted with caution. Future studies will include larger independent cohorts to validate these modules’ reliability. This study used blood samples for their accessibility, facilitating clinical translation. However, gene expression changes in blood may reflect systemic alterations rather than the specific intracranial pathological processes of PD. Caution is needed when directly linking these findings to central lysosomal dysfunction in PD. That said, considering the peripheral-central interplay characteristic of PD pathology—such as peripheral immune abnormalities, like increased monocyte infiltration, potentially crossing the blood-brain barrier and exacerbating central microglial activation and neuroinflammation, and gut inflammation transmitting peripheral signals to the central nervous system through the gut-brain axis, affecting lysosomal-autophagy function (Morris et al., 2024)—the observed overexpression of *FGD4* and *MAN2B1* in blood may indirectly contribute to central inflammatory pathology in PD by modulating peripheral monocyte infiltration. Alternatively, they may serve as surrogate markers reflecting the systemic pathological state of PD. To further clarify this, future studies will involve correlating *FGD4* and *MAN2B1* expression in both brain tissue and peripheral blood using PD animal models to determine how well peripheral biomarkers reflect central pathology. While this study has computationally identified and experimentally validated *FGD4* and *MAN2B1* as lysosome-related biomarkers for PD, and preliminary PCR experiments have investigated their expression trends in clinical samples, the functional mechanisms remain to be explored. Further cellular functional assays, such as gene overexpression/knockdown using techniques like siRNA or CRISPR, will be conducted to assess their specific roles in PD pathogenesis, including their impact on core PD pathological markers such as lysosomal pH. Additionally, co-immunoprecipitation (Co-IP) will be performed to determine whether direct PPIs exist between *FGD4*, *LRRK2*, and *COMT*. PD animal models will also be developed to evaluate *FGD4* and *MAN2B1* expression, with an emphasis on FOG-like behaviors, to assess whether *FGD4* and *MAN2B1* influence PD progression and FOG phenotypes through regulation of lysosomal function or FOG-related genes like *LRRK2* and *COMT*. Moreover, leveraging the validated gene associations, the findings of this study may have potential clinical applications in diagnostic panels. Large-scale prospective cohort studies are needed to validate these diagnostic models, which could incorporate multi-gene blood test panels featuring *FGD4*, *MAN2B1*, and other novel markers. Depending on clinical scenarios, methods like qPCR or digital PCR may be applied for non-invasive early screening.

## 5 Conclusion

In conclusion, *FGD4* and *MAN2B1* function as lysosomal biomarkers associated with PD and show significant correlations with genes linked to PD-related freezing of gait. This study offers new perspectives for the diagnosis and understanding of PD.

## Data Availability

The data presented in the study are publicly available in FigShare at https://doi.org/10.6084/m9.figshare.31078831.
