# Supplementary figures and images for: RNA networks of lysosomal-related biomarkers in Parkinson’s disease and their correlations with freezing of gait-associated genes
Source: Front Genet. 2026 Jan 28;17:1632163. doi: 10.3389/fgene.2026.1632163 (PMC12890354; doi:10.3389/fgene.2026.1632163)

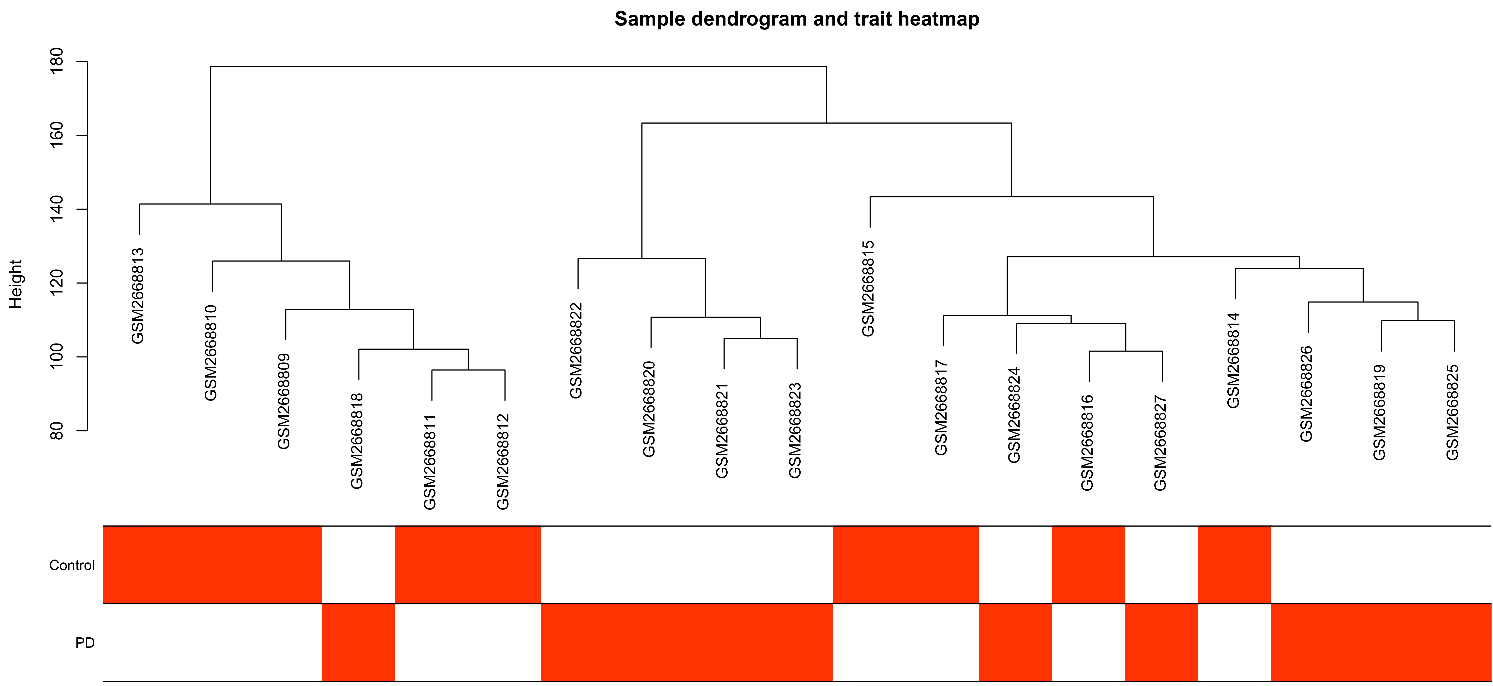


**Supplementary Figure 1.** Critical modules related to PD selected by WGCNA

Supplement: Supplementary file 12 [file DataSheet1.docx]
